# Isolated IgG4-related sclerosing cholangitis misdiagnosed as malignancy in an area with endemic cholangiocarcinoma: a case report

**DOI:** 10.1186/s12893-017-0214-1

**Published:** 2017-02-15

**Authors:** Narongsak Rungsakulkij, Pattana Sornmayura, Penampai Tannaphai

**Affiliations:** 1 Department of Surgery, Ramathibodi Hospital, Mahidol University, Bangkok, 10400 Thailand; 2 Department of Pathology, Ramathibodi Hospital, Mahidol University, Bangkok, 10400 Thailand; 3 Department of Radiology, Ramathibodi Hospital, Mahidol University, Bangkok, 10400 Thailand

**Keywords:** IgG4, IgG4-related sclerosing cholangitis, Cholangiocarcinoma, Benign biliary stricture, Sclerosing cholangitis

## Abstract

**Background:**

The most common cause of perihilar obstruction is cholangiocarcinoma, especially in Thailand. Benign perihilar stricture represents less than 20% of cases. IgG4-related disease and IgG4-related sclerosing cholangitis, however, have been receiving increased recognition. Isolated IgG4-related sclerosing cholangitis is less common. The preoperative diagnosis of IgG4-related sclerosing cholangitis without pancreatic involvement is very difficult because the clinical presentation and preoperative evaluation are extremely difficult to distinguish from perihilar cholangiocarcinoma.

**Case presentation:**

We report the case of a 56-year-old man who presented with obstructive jaundice with preoperative imaging showing proximal common bile duct obstruction. He underwent right lobe liver hepatectomy with extrahepatic bile duct resection and regional lymph node dissection due to high suspicion of malignancy. The pathological report showed severe acute and chronic inflammation of the bile duct with morphology and immunohistochemistry suggestive of IgG4-related sclerosing cholangitis.

**Conclusions:**

IgG4-related sclerosing cholangitis with perihilar obstruction should be considered even in areas where cholangiocarcinoma is endemic.

## Background

Thailand has the highest incidence of cholangiocarcinoma (CCA) worldwide, especially in the northeast area [1]. Malignant appearing perihilar obstruction is commonly associated with CCA until proven otherwise [2]. The incidence of benign obstruction reported in literature ranges from 8–17.4% [2–13]. IgG4-related disease (IgG4-RD) is an emerging entity increasingly described in the literature. Autoimmune pancreatitis is the most common organ involvement [14–16]. IgG4-related sclerosing cholangitis (IgG4-SC) is reported worldwide in Western and Asian populations [5, 11–13]. Distal biliary stricture is the most common presentation in association with autoimmune pancreatitis (AIP). However, IgG4-SC with proximal perihilar biliary obstruction in the absence of AIP is uncommon [12]. The clinical manifestation is similar to CCA, especially for proximal biliary stricture. Diagnosis can be established by clinical suspicion with diagnosis criteria [16–18]. Steroid treatment should be empirically provided in suspected cases [18]. A minority of cases have been managed with surgical resection, usually in the case of isolated biliary strictures. We report a case of IgG4-SC in the absence of AIP that was preoperatively misdiagnosed as CCA, and the patient underwent right hemihepatectomy with bile duct resection and biliary enteric reconstruction. The pathological report showed lymphoplasmacytic infiltration with IgG4-positive plasma cells. The ultimate diagnosis was IgG4-associated cholangitis.

## Case presentation

A 56-year-old Asian male patient from northeast, Thailand with an unremarkable past-medical history was referred from a primary care center with painless obstructive jaundice for two weeks. He was previously admitted to a private hospital, and abdominal computed tomography (CT) was performed. He underwent endoscopic retrograde cholangiopancreatography (ERCP) and trans-ampullary biopsy and brush cytology. A plastic biliary stent was inserted across the obstruction point. At the time of the initial visit to the out-patient clinic, physical examination revealed a mildly obese patient, generally co-operative, with marked icteric sclera and pruritus. No acute distress was noted. There were no clinical signs of an abdominal mass or palpable supraclavicular lymph node.

At the time of referral, laboratory analysis showed the following liver function test results: total bilirubin 16.0 mg/dL, direct bilirubin 11.7 mg/dL, alkaline phosphatase (ALP) 319 U/L (normal 50–136 U/L), gamma glutamyl transferase (GGT) 33 U/L (normal 15–85 U/L), aspartate aminotransferase (AST) 59 U/L (normal 15–37 U/L), alanine aminotransferase (ALT) 55 U/L (normal 30–65 U/L), white blood cell count 12,040/cumm, platelet count 464,000/cumm, hemoglobin 11.5 g/L, CEA 1.9 ng/ml (0–4.6 ng/mL), CA 19–9 19.8 U/mL (normal 0–39 U/mL), normal serum electrolytes, normal renal function. HBsAg, anti-HCV and anti-HAV were all negative.

**Fig. 1:**
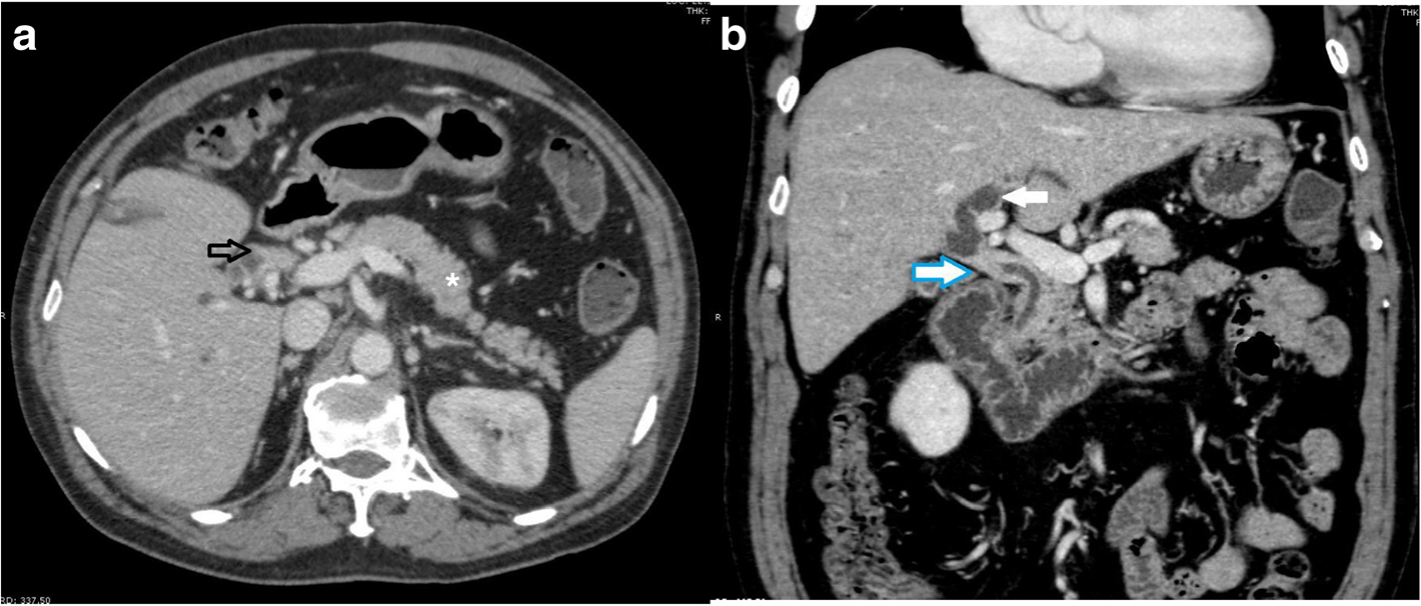
Abdominal CT showing focal short segment sheet-like thickening of proximal common bile duct with enhancement. **a**: axial view showing sheet-like thickening of common hepatic duct wall (*arrow*); note the normal pancreas (*star*). **b**: coronal view showing focal short segment, sheet-like, symmetric thickening of common hepatic duct causing nearly complete obstruction of common hepatic duct just below bifurcation (*white arrow with blue edge*) of right and left intrahepatic bile ducts and dilatation of left intrahepatic duct (*white arrow*)

**Fig. 2:**
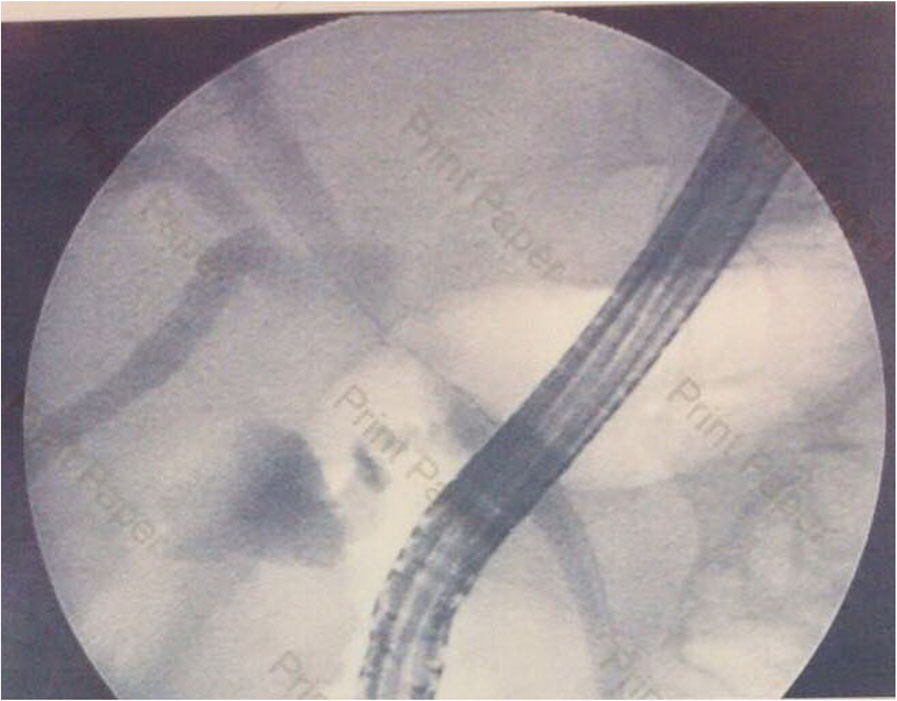
ERCP show stricture of common hepatic duct just below hepatic confluence

Transabdominal ultrasonography was performed at a private hospital and showed dilatation of both intrahepatic bile ducts. The common bile duct was not dilated. No gallstone was found. Abdominal CT showed a focal short segment, sheet-like, symmetric thickening of the common hepatic duct causing nearly complete obstruction just below the bifurcation of the right and left intrahepatic bile ducts and dilatation of the left intrahepatic duct (Fig. 1). At private hospital, he underwent ERCP which revealed a common hepatic duct stricture of approximately 1–2 cm just below the biliary confluence and dilatation of both the right and left intrahepatic bile ducts (Fig. 2). Brush cytology and trans-ampullary biopsy were performed. The cytological reports and trans-ampullary biopsy pathological reports were benign bile duct epitheliums without cellular atypia.

At the time of referral, chest and mediastinum CT were performed, and the results showed no pulmonary metastasis or new lesions, along with a decreased degree of intrahepatic bile duct dilatation. Magnatic resonance imaging (MRI) and magnetic resonance cholangiopancreatography was not done in this patient. Ultimately, surgical resection was planned after adequate biliary drainage due to high suspicion of CCA.

**Fig. 3:**
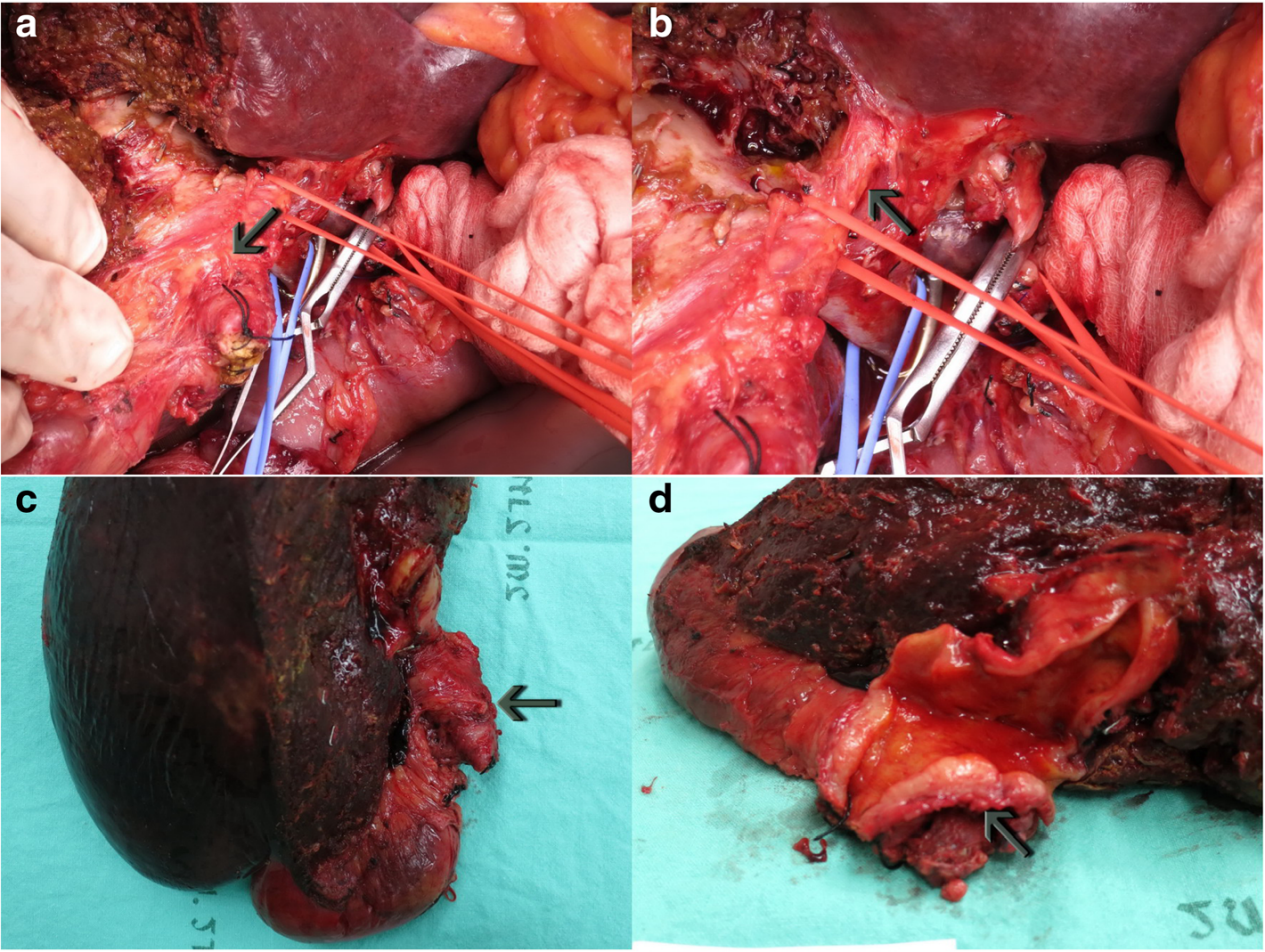
Intraoperative findings and gross specimen. **a**: tumor at hepatic hilum (*arrow*). **b**: left main intrahepatic duct (*arrow*). **c**: gross specimen after complete resection showing mass at proximal common bile duct just below hepatic confluence (*arrow*). **d**: diffuse bile duct thickening with marked focal thickening at common hepatic duct (*arrow*)

At surgery, the abdomen was opened by inverted J skin incision. There was no metastatic disease. There was a hard palpable lesion at the common bile duct along the common hepatic duct (Fig. 3). Extended right hepatic lobectomy with en-bloc excision of the gallbladder and extrahepatic bile duct with lymphadenectomy was performed, followed by reconstruction using Roux-en-Y hepatico-jejunostomy anastomosis to the left intrahepatic bile duct. Intraoperative frozen sections of the resection margins of the distal common bile duct were negative for malignancy.

**Fig. 4:**
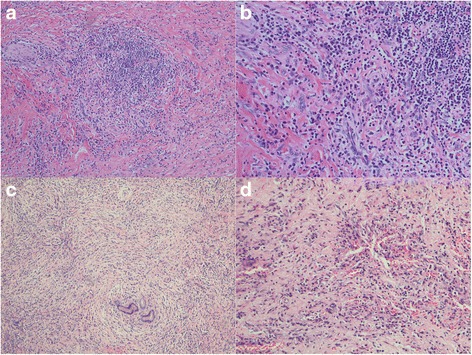
Left hepatic duct along with common bile duct showing fibrosis and plasma cells. **a**, H&E x100; **b**. H&E x200; **c**, H&E x100 storiform fibrosis with lymphocytes and plasma cells; **d**, H&E x200 obliterative phlebitis H&E, Hematoxylin and eosin stain

**Fig. 5:**
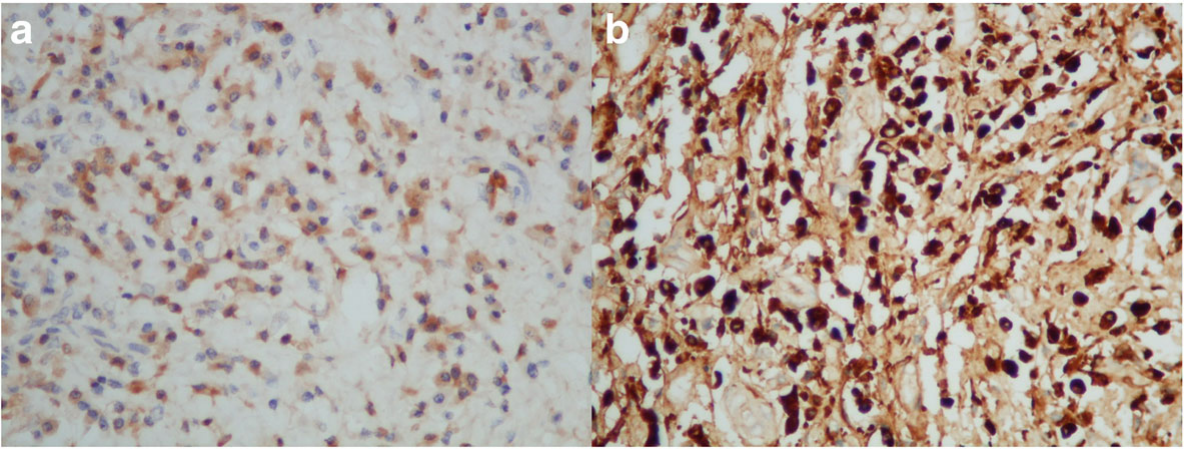
Left hepatic duct along with common bile duct showing positive plasma cells. **a**. IHC x400 of IgG-positive plasma cells. **b**. IHC x400 of IgG4-positive plasma cells (80 cells/HPF) IHC, immunohistochemistry

The pathological examination of the surgical specimen revealed severe acute and chronic inflammation with dense lymphoplasmacytic infiltration, storiform fibrosis and obliterative phlebitis of the common hepatic duct and right and left hepatic ducts, with all margins inflamed (proximal bile duct margin, distal bile duct margin and hepatic parenchymal margin) as shown in Fig. 4. All lymph nodes including the celiac, common hepatic, retropancreatic and hepatoduodenal lymph node groups were negative for malignancy. After review of the pathological specimens by a pathologist, we requested further immunohistochemical analysis for IgG4-related disease, and the results showed IgG4-positive plasma cells at 80 cells/HPF (Fig. 5) for the bile duct wall, with the lymph node showing mixed follicular and paracortical lymphoid hyperplasia with markedly increased polytypic plasma and an IgG4/IgG ratio above 40%.

Postoperatively, the patient suffered from intra-abdominal infection from hepatico-jejunostomy anastomosis leakage that required re-laparotomy and repair of the anastomosis and abdominal toilet. After the re-laparotomy, he recovered uneventfully. The serum IgG4 level obtained after surgery was above 7230 mg/dL. At 6 months follow-up, he had recovered well and maintained normal activity with no recurrence of the disease. His laboratory analysis results were normal, and abdominal CT showed no other biliary stricture. There is no further treatment after discussing with gastroenterologist.

## Discussion

The most common cause of perihilar biliary obstruction is perihilar CCA (Klatskin tumors) [2], especially in Thailand, which has the highest prevalence of both intrahepatic and extrahepatic CCA worldwide due to parasitic infestation [1]. Surgical resection including major hepatic resection remains the mainstay of treatment for perihilar CCA [19]. According to the AJCC classification 7^th^ edition, the extrahepatic bile duct is divided into the perihilar (proximal common bile duct and hilar) and distal common bile duct [20]. The incidence of benign proximal biliary obstruction preoperatively diagnosed as hilar CCA in which the final histological examination of resection specimens shows benign stricture is approximately 8–17.3% [2–13]. One type of benign perihilar stricture is IgG4-RD. IgG4-RD is an emerging systemic condition characterized by mass-forming sclerosing lesions, elevated serum IgG4 concentrations, and extensive tissue infiltration by IgG4-positive plasma cells. IgG4-RD can involve any organ, but the most common is the pancreas. IgG4-SC is the sixth most common type of organ involvement [14]. IgG4-SC is a characteristic type of sclerosing cholangitis with dense infiltration of IgG4-positive plasma cells and extensive fibrosis in the bile duct wall [17]. IgG4-SC concomitant with pancreatic involvement or AIP is the most common presentation, while isolated IgG4-SC is uncommon. The previously reported incidence of perihilar stricture due to IgG4-SC without pancreatic involvement represents 4.8–9.1% of benign biliary stricture [12]. Zen et al. reported the prevalence and incidence of proximal IgG4-SC to be 1.0 and 0.3 per 100,000 population, respectively [17].

The diagnosis of IgG4-SC is crucial. Because IgG4-SC lacks any highly specific diagnostic features, a high index of suspicion in all patients with unexplained biliary strictures can lead to the most accurate diagnosis. Some authors have proposed criteria for the diagnosis of IgG4-SC. Firstly, Ghazale et al. from the Mayo group proposed the worldwide acceptance of the HISORt criteria for IgG4-SC, adopted from previously reported criteria for AIP including histology of the bile duct, imaging of the bile duct, serology, other organ involvement and response to steroid therapy [15, 18]. Recently, Ohara et al. reported Japanese and Korean guidelines for the clinical diagnostic criteria of IgG4-related sclerosing cholangitis [21]. According to these guidelines, the current case was definitely diagnosed with IgG4-SC. The most important clue to diagnosis were three distinct histopathological findings including markedly dense fibrous tissue in the background with focal storiform pattern and densely infiltrating lymphocytes and plasma cells and obliterative phlebitis. The IgG4+/IgG+ plasma cell ratio was more than 40% and IgG4+ plasma cells density was 80cells/HPFs [18, 21].

Serum IgG4 level is an important part of the diagnosis of IgG4-SC. However, an increased serum IgG4 level alone should not be used to diagnose of IgG4-SC. Ghazale et al. report the sensitivity of serum IgG4 to be 74% [18]. Oseini et al. report that a cut-off level of serum IgG4 above 135 or 140 mg/dl shows higher sensitivity, while a serum IgG4 level 4-fold higher than normal is 100% specific for IgG4-SC [22]. Lytras et al. report the sensitivity and specificity of serum IgG4 level to be 50% and 75%, with the positive predictive value (PPV) and negative predictive value (NPV) of 75% and 50%, respectively. The accuracy of elevated serum IgG4 for the diagnosis of IgG4-SC reached 69% [13]. Ohara et al. report a large cohort study from Japan that increasing specificity for preoperative diagnosis by cytology of hilar involved IgG4-SC to 96.6% when a cut-off level of serum IgG4 level is 207 mg/dL [23].

Cross-sectional study, CT and MRI have proven difficult to use for differentiation from CCA [24–27]. IgG4-SC can present with a focal/segmental or diffused biliary stricture [26, 27]. Patients with concomitant AIP and other organ involvement might demonstrate characteristics of organ involvement that raise suspicions of IgG4-SC. The absence of other organ involvement could make it more difficult for a physician to distinguish IgG4-SC from CCA. Some authors have described image findings for the diagnosis of IgG4-SC including multifocal biliary strictures, a markedly thickened bile duct wall (mean wall thickness, 4.9 mm), a smooth outer margin, a narrow but visible lumen, hyper-enhancement during the late arterial phase, homogeneous hyper-enhancement during the delayed phase, concurrent gallbladder wall thickening and an absence of vascular invasion [27–29].

Recently, Chen L et al. have reported the accuracy rate, sensitivity, specificity, PPV and NPV of intraductal ultrasound (IDUS) with tissue sampling in proximal bile duct obstruction to be above 90% [30]. The characteristic IDUS findings for IgG4-SC are circular symmetrical wall thickness, a smooth outer margin, a smooth inner margin and a homogeneous internal echo in the stricture [31].

Isolated IgG4-SC without AIP is uncommon. Despite aggressive preoperative evaluation, especially in the center that have limitation of resource, it is very difficult to differentiate IgG4-SC without AIP from CCA. Preoperative histology in biliary stricture remains controversial. Davidson et al. concluded that there is currently no evidence to support routine histological examination for the diagnosis of CCA [32]. But his study does not employ the results of a number of studies that have demonstrated the value of histology, the role of additional studies such as fluorescence in situ hybridization (FISH). Recently, the incorporation of routine cytology and FISH for evaluation of endoscopic brushings would be an important role for diagnosis of pancreatobiliary tract malignancy [33]. Some reported cases of IgG4-SC in the absence of AIP have been misdiagnosed as cholangiocarcinoma and treated by surgical resection (Table 1). In this case, the misdiagnosis was partly because CCA is very common in Thailand. The clinical manifestation was similar to CCA. Surgical resection was recommended to these patients because of high suspicion of CCA that could not be distinguished from benign biliary stricture. According to current available guidelines, some authors recommend steroid treatment and waiting for response to therapy [18, 21].

**Table 1:** Review of reported cases of isolated IgG4-associated cholangitis misdiagnosed as perihilar cholangiocarcinoma underwent surgical resections

Author/ Year	N	Sex	Age	Stricture location/Symptom	IgG4 (mg/dL)	Operation	Pathology
Hamano (2005) [34]	1	M	56	Perihilar, CBD/ Jaundice	1646	Hepatic resection with pancreaticoduodenectomy	Lymphoplasmacytic infiltration IgG4-positive plasma cell infiltration
Cheung (2008) [35]	1	F	68	Perihilar/ Jaundice	2890	Hepatic resection	Lymphoplasmacytic sclerosing cholangitis IgG4-positive plasma cells
Erdogan (2008) [11]	2	NA	NA	NA/ Jaundice	NA	Hepatic resection	Lymphoplasmacytic infiltration Moderate IgG4-positive plasma cells
Fujita (2010) [12]	1	M	60	Perihilar/ Jaundice	NA	Bile duct resection	Lymphoplasmacytic infiltration Diffuse IgG4-positive plasma cells
Lytras (2012) [13]	1	NA	NA	NA/ NA	Negative	Hepatic resection	Class A (HISORt criteria)
Nguyen-tat (2012) [36]	1	M	79	Perihilar/ Jaundice	40	Hepatic resection with bile duct resection	Lymphoplasmacytic infiltration IgG4-positive plasma cells > 50 cells/HPF
Matusubayashi (2014) [37]	1	M	70	Perihilar/ Jaundice	1.10	Hepatic resection with bile duct resection	Lymphoplasmacytic infiltration IgG4-positive cells 10 cells/HPF
Zaydfudim (2015) [38]	2	M M	68 79	Perihilar/ abdominal pain Perihilar/ abnormal LFT	61.3 39.4	Hepatic resection with bile duct resection Hepatic resection with bile duct resection	Lymphoplasmacytic infiltration IgG4-positive plasma cells > 40% IgG4-positive plasma cells >50 cells/HPF
Miki (2015) [39]	1	M	69	Perihilar/ Jaundice	381	Bile duct resection	Lymphocyte and plasma cell infiltration IgG4-positive plasma cells > 50 cells/HPF
Ignjatovic (2015) [40]	1	M	60	Perihilar, CBD/ Jaundice, abdominal pain	80	Bile duct resection	Dense fibro-inflammatory infiltration with lymphoid cells IgG4-positive plasmacytoid cells
Lin (2015) [41]	4	M M M M	84 70 72 61	Perihilar/ Jaundice	NA	Bile duct resection	Lymphoplasmacytic infiltration IgG4-positive cells >50 cells/HPF
Present study	1	M	56	Perihilar/ Jaundice	>7230	Hepatic resection with bile duct resection	Lymphoid and plasma cell infiltration IgG4-positive plasma cells 80 cells/HPF

*M* male, *F* female, *CBD* common bile duct, *LFT* liver function test, *HPF* high-power field

## Conclusions

In conclusion, surgeons should be aware of IgG4-RD as one of the causes of benign hilar and proximal bile duct stricture and consider this possibility preoperatively. The serum IgG4 level and associated other organ involvement should be investigated. However, the clinical presentation and imaging study are extremely difficult to distinguish from malignancy. A high index of suspicion and consideration of diagnostic criteria are important. The accurate diagnosis of IgG4-SC is important to avoid major surgical resection in patients with perihilar biliary obstruction. More invasive studies such as ERCP with IDUS and EUS show promising results in preoperative diagnosis.

## Abbreviations

AIP: Autoimmune pancreatitis
ALP: Alkaline phosphatase
ALT: Alanine aminotransferase
AST: Aspartate aminotransferase
CA 19–9: Cancer antigen 19–9
CCA: Cholangiocarcinoma
CEA: Carcinoembryonic antigen
CT: Computed tomography
ERCP: Endoscopic retrograde cholangiopancreatography
GGT: Gamma glutamyl transferase
HAV: Hepatitis A virus
HBsAg: Hepatitis B surface antigen
HCV: Hepatitis C virus
IDUS: Intraductal ultrasound
IgG4-RD: IgG4-related disease
IgG4-SC: IgG4-sclerosing cholangitis
NPV: Negative predictive value
PPV: Positive predictive value
SOPC: Single-Operator Peroral Cholangioscopy
